# Supplementary material for: Radiomics Analysis in Characterization of Salivary Gland Tumors on MRI: A Systematic Review
Source: Cancers (Basel). 2023 Oct 10;15(20):4918. doi: 10.3390/cancers15204918 (PMC10605883; doi:10.3390/cancers15204918)
Supplement: Supplementary file 1 [file cancers-15-04918-s001.zip › cancers-2634640-supplementary.pdf]

Supplementary Materials: Radiomics Analysis in Characterization of Salivary Gland Tumors on MRI: A Systematic Review

Kaijing Mao, Lun M. Wong, Rongli Zhang, Tiffany Y. So, Zhiyi Shan, Kuofeng Hung and Qiyong H. Ai

Table S1. Details of radiomics protocols used in each included study.

Study ID	Image acquisition and segmentation				Feature extraction and selection		Model	
	Investigated sequence(s)	ROI/VOI	Segmentation	Intra-observer (ICC)	Features selection method	Feature Validation	Classifier	Classifier Validation
1	DWI	Whole tumor	manual	> 0.75	PCC value, RFE algorithm	5-fold cross-validation	LDA	internal validation
2	T2WI	Whole tumor	manual	NA	Wilcoxon rank-sum test, PCC value	NA	SVM	NA
3	T1WI, fs-T2WI	Whole tumor	Manual	> 0.75	LASSO, ANOVA	10-fold cross-validation	LR	external validation
4	T1WI, T2WI CE-T1WI	Two slices	Manual/ automatic	NA	An operator-independent statistical system	NA	LR	cross-validation
5	DWI	VOI whole tumor	Manual	>0.75	LASSO, ANOVA	5-fold cross-validation	LR, SVM, KNN	cross-validation
6	T1WI, T2WI CE-T1WI	Whole tumor	Manual	>0.75	LASSO, The Select K Best	10-fold cross-validation	XGBoost, SVM, DT	internal validation
7	T1WI, T2WI	Whole tumor	Semiautomatic	NA	PCC	Bootstrap	NA	internal validation
8	T1WI, fs-T2WI	Whole tumor	Manual	>0.75	LASSO, ANOVA	10-fold cross-validation	NA	external validation
9	T1WI; CE-T1WI T2WI, DWI, DCE	Whole tumor	Manual	NA	AIC, BIC	Leave-one-out cross-validation	LDA, SVM	Cross-validation
10	T1WI T2WI	NA	Manual	>0.75	LASSO, t-test, Mann–Whitney U test	10-fold cross-validation	LR, SVM	internal validation
11	T1WI, fs-T2WI CE-T1WI	NA	Manual	NA	LASSO, t-test, Wilcoxon rank-sum test	10-fold cross-validation	LR	cross-validation
12	T1WI, T2WI	NA	Automatic	NA	LASSO	10-fold cross-validation	NA	NA
13	T2WI	Whole tumor	Manual	>0.75	LASSO, t-test	5-fold cross-validation	LR	internal validation
14	T2WI	Whole tumor	Manual	NA	Kruskal–Wallis test	NA	LR, SVM, NNET DT	internal validation

AIC: Akaike Information Criterion, ANOVA: One-way analysis of variance, BIC: Schwarz Bayesian Information Criterion, CE: contrast enhanced, DCE: dynamic contrast enhanced, DT: Decision tree, DWI: diffusion-weighted imaging, FS: fat saturation, ICC: intra-class correlation coefficient, KNN: K-nearest neighbor, LASSO: least absolute shrinkage and selection operator, LDA: linear discriminant analysis, LR: logistic regression, NA: not available, NNET: artificial neural network, PCC: Pearson correlation coefficient, RFE: recursive feature elimination, ROI/VOI: region/volume of interest, SVM: support vector machine, T1WI: T1 weighted image, T2WI: T2 weighted image, XGBoost: Extreme gradient boosting.

Table S2. QUADAS-2 assessment for each study.

Study	RISK OF BIAS				APPLICABILITY CONCERNS		
	PATIENT SELECTION	INDEX TEST	REFERENCE STANDARD	FLOW AND TIMING	PATIENT SELECTION	INDEX TEST	REFERENCE STANDARD
Study 1	☺	☺	☺	?	☺	☺	☺
Study 2	?	☺	☺	?	☺	☺	☺
Study 3	☺	☺	☺	?	☺	☺	☺
Study 4	☺	☺	☺	?	☺	☺	☺
Study 5	☺	☺	☺	?	☺	☺	☺
Study 6	☺	☺	☺	?	☺	☺	☺
Study 7	☺	☺	☺	?	☺	☺	☺
Study 8	☺	☺	☺	?	☺	☺	☺
Study 9	?	☺	☺	?	☺	☺	☺
Study 10	?	☺	☺	?	☺	☺	☺
Study 11	?	☺	☺	?	☺	☺	☺
Study 12	☺	☺	☺	?	☺	☺	☺
Study 13	☺	☺	☺	?	☺	☺	☺
Study 14	?	☺	☺	?	☺	☺	☺

☺Low Risk ☹High Risk ? Unclear Risk; QUADAS-2: Quality Assessment of Diagnostic Accuracy Studies-2.

Table S3. Individual and summarized RQS scores for each study.

Study ID	Image protocol	Multiple segmentations	Phantom study on all scanners	Multiple time points	Feature reduction	Non-radiomics features	Biological correlates	Cut-off	Discrimination	Calibration	Prospective study	Validation	Comparison to gold standard	Potential clinical utility	Cost-effectiveness analysis	Open science	Total
1	1	1	0	0	3	0	0	0	2	0	0	2	2	0	0	0	11
2	1	0	0	0	3	0	1	0	1	0	0	2	2	2	0	0	12
3	1	1	0	0	3	1	0	0	1	1	0	3	2	2	0	0	15
4	1	0	0	0	3	0	1	0	1	0	0	2	2	2	0	0	12
5	1	1	0	0	3	0	1	0	2	0	0	2	2	0	0	0	12
6	1	1	0	0	3	0	0	0	2	0	0	2	2	2	0	0	13
7	1	0	0	0	3	1	1	0	1	1	0	2	2	2	0	0	14
8	1	1	0	0	3	1	1	0	1	1	0	3	2	2	0	0	16
9	1	0	0	0	3	1	0	0	2	0	0	2	2	0	0	0	11
10	1	1	0	0	3	0	0	0	1	1	0	2	2	2	0	0	13
11	1	0	0	0	3	0	0	0	2	0	0	2	2	0	0	0	11
12	1	0	0	0	3	1	1	0	1	1	0	2	2	2	0	0	14
13	1	1	0	0	3	1	0	0	1	1	0	2	2	2	0	0	14
14	1	1	0	0	3	1	1	0	1	0	0	2	2	0	0	0	12

RQS: radiomics quality score.
